# Intestinal Microbiota as Modulators of the Immune System and Neuroimmune System: Impact on the Host Health and Homeostasis

**DOI:** 10.1155/2015/931574

**Published:** 2015-02-22

**Authors:** Carlos Magno da Costa Maranduba, Sandra Bertelli Ribeiro De Castro, Gustavo Torres de Souza, Cristiano Rossato, Francisco Carlos da Guia, Maria Anete Santana Valente, João Vitor Paes Rettore, Claudinéia Pereira Maranduba, Camila Maurmann de Souza, Antônio Márcio Resende do Carmo, Gilson Costa Macedo, Fernando de Sá Silva

**Affiliations:** ^1^Department of Biology, Institute of Biological Sciences, Federal University of Juiz de Fora, 36036-900 Juiz de Fora, MG, Brazil; ^2^Department of Pharmacy, Federal University of Juiz de Fora, Governador Valadares, Brazil; ^3^Department of Immunology, University of São Paulo, São Paulo, Brazil; ^4^Department of Nutrition, Federal University of Juiz de Fora, Governador Valadares, Brazil; ^5^Department of Clinical Dentistry, Federal University of Juiz de Fora, Juiz de Fora, Brazil; ^6^Department of Parasitology, Microbiology and Immunology, Federal University of Juiz de Fora, Juiz de Fora, Brazil; ^7^Department of Dentistry, Federal University of Juiz de Fora, Governador Valadares, Brazil

## Abstract

Many immune-based intestinal disorders, such as ulcerative colitis and Crohn's disease, as well as other illnesses, may have the intestines as an initial cause or aggravator in the development of diseases, even apparently not correlating directly to the intestine. Diabetes, obesity, multiple sclerosis, depression, and anxiety are examples of other illnesses discussed in the literature. In parallel, importance of the gut microbiota in intestinal homeostasis and immunologic conflict between tolerance towards commensal microorganisms and combat of pathogens is well known. Recent researches show that the immune system, when altered by the gut microbiota, influences the state in which these diseases are presented in the patient directly and indirectly. At the present moment, a considerable number of investigations about this subject have been performed and published. However, due to difficulties on correlating information, several speculations and hypotheses are generated. Thus, the present review aims at bringing together how these interactions work—gut microbiota, immune system, and their influence in the neuroimmune system.

## 1. Introduction

The human body is colonized by a vast number of microbes, collectively referred to as the human microbiota. The link between these microbes and our health is the focus of a growing number of research initiatives, and new insights are emerging rapidly. The fact that the number of microbial cells composing the human microbiota surpasses that of own body cells allows us to foresee the existence of an intertwined relationship between the biology of the human host and such microorganisms, which has been moulded by millennia of evolution. Studies regarding the understanding of the various aspects of the conjunct of unicellular organisms carried in the human body rely on molecular biology tools in order to unravel the species that are present as well as the genes found to be operating the host-microorganism interaction [1]. Over the past few years, next-generation DNA sequencing has allowed substantial fulfilment of the efforts directed at clarifying aspects related to our whole microbiota, concerning mainly its composition and the inherent variability, which may occur interpersonally and in a single individual in the course of one day or due to aging. Besides, the cited variability may occur as a response to certain illnesses; taking advantage of it, this variability can constitute a powerful diagnostic tool and give important clinical correlations [2–4].

Considering that humans, as well as other multicellular organisms, have evolved in an environment where unicellular organisms have always been ubiquitous, it is intuitive to think that the composing elements of our microbiome started to be selected much earlier in our evolutionary history. The implication is that both our metabolic traits and those of the organisms we host have been forged by evolution in a mutualistic fashion, so that the presence of certain microorganisms is connected to physiological functioning, and variations of the microbial composition of our bodies may be linked to metabolic alterations in various sites on the human body [5]. Here, we are going to focus on the alterations that may occur in the gut microbiota.

Gut microbiota gives individual-specific milieu for ingested food, and host intestine provides unique genetic background for the growth of specific bacteria. The human gastrointestinal tract is inhabited by 1 × 10^13^ to 1 × 10^14^ microorganisms and from 500 to 1,000 species [6, 7] and more than 7,000 strains [8]. The balance between this complex community of gut bacteria, food nutrients, intestinal genomics, and physiological site is increasingly recognized as a major contributor to human health. In certain disorders where environmental factors are implicated, an imbalance between commensal bacteria with pathogenic potential (which we term pathobionts) and commensal bacteria with beneficial potential (symbionts) has a role in pathogenesis.

Arumugam et al. [26] have highlighted the advances made on understanding the gut microbiota by summarizing and adding data from metagenomic sequencing of stool samples. The intestinal microbiota has bacteria as its virtually sole component. Bacteroidetes, which is an abundant phylum, together with Firmicutes, correspond to 90% of the intestinal gut pool of microorganisms [6]. There are also efforts to determine the enterotypes: clusters in which the levels of three genera among the whole gut microbiome varied in a similar way. Enterotype 1 was identified by the variation of* Bacteroides* and enterotype 2 displayed altered levels of* Prevotella*, both of them components of the Bacteroidetes phylum; and enterotype 3,* Ruminococcus*, belongs to the Firmicutes phylum. These enterotypes have been shown to be highly robust and were not restricted to region, country, or continent. The bacterial genera enriched in each of the enterotypes appear to be connected to the mechanism by which the intestinal microbiota degrades fermentable substrates in the colon [26]. Enterotypes clusters depend on long-term diets whose changes can be detectable 24 hours after diet change and remained stable after 10 days [27]. However different, the enterotypes could not be connected to any of the host features measured nationality, gender, age, or body mass [26]. Nevertheless, de Fellipo et al. have found that African children who have diet high in fiber compared to European children showed a significant enrichment in Bacteroidetes and depletion in Firmicutes as well as the microbial biodiversity [28].

Although the gut microbiome is highly variable, the summation of genomes comprised in it tends to be quite conserved when considered the microbial metabolic pathways [29], being particularly relevant when discussing the gut microbiome, in order to understand the underlying mechanisms involved in the host-microbiota relationship both in healthy individuals and in those suffering from intestinal or metabolic diseases [3, 30].

The microbial metabolism is seen as a complement to the host metabolism. Thereby, alterations in this metabolism, due to alterations either in microbiota composition or in diet or some other modifications, can happen and have been specifically related to diseases, among which are irritable bowel syndrome (IBS) [9–11]; inflammatory bowel disease (IBD), as ulcerative colitis and Crohn's disease [12–14]; colorectal cancer [15, 16]; obesity [31]; type 1 diabetes [32]; and type 2 diabetes [18].

Although those are multifactorial conditions, they seem to be connected to the intestinal microbiota, in a relationship not yet fully understood. Studies showed that altered balance between the two major enteric bacterial phyla, the Bacteroidetes and the Firmicutes, has been associated with clinical states, and microbial and nutrient lifetime changes, from early metabolic programming to late age immunity decline, may have major impact on health and well-being [33]. The microbial alterations apparently involved in the pathogenesis of some specific diseases are displayed in Table 1.

Concerned with finding answers to understand the connection of gut microbiota and disease, studies have already perceived the influence of human gut microbiota and its perturbations on homeostasis, as already cited, nutrition and behaviour, due to the connection of these microbes to the availability of nutrients, and modulation of the immune, neuronal, and endocrine systems [34, 35]. Thus, the gut microbiota in fact participates in the regulation of physiological and metabolic pathways. In the next topics, the major and current interactions of gut microbiota and other systems will be related. All metabolic and physiologic forms of alterations influenced by the gut microbiota or influencing its composition reflect systemic-wide alteration of balance. The best-described host-microbiota interaction to date is that involving the intestinal epithelium and the immune system, with increasing knowledge about neuroimmune interaction.

## 2. Gut Microbiota and Immune System

The human gastrointestinal tract is constantly in contact with an overwhelming antigenic load in the form of commensal bacteria and dietary antigens. The system must be able to discriminate pathogens that require a protective immune response, from normal microbiota or food antigens, where a dynamic unresponsiveness state is necessary [36].

The gastrointestinal tract (GI) is inhabited by several types of microorganisms (bacteria, virus, protozoan, etc.)—the gut microbiota. Commensal bacteria, the most frequent microorganisms in intestinal environment, are beneficial for the host, while pathogenic bacteria are able to cause problems, such as gut inflammation and invasiveness. The symbiosis process happens when there is a favourable balance between commensal bacteria and pathogenic bacteria over a period of time [37]. In this process, the interaction of microbiota, intestinal epithelium, and mucosal immune system results in a local and systemic homeostasis. However, in a dysbiosis process, the interaction between commensal and pathogenic bacteria is altered, resulting in homeostasis disruption [38]. This breakdown of homeostasis can result from local infection and inflammation to complications that can affect several other human systems like the central nervous system and endocrine system [39]. In the next paragraphs, we will describe, briefly, how intestinal immune system is formed and how it interacts with microbiota.

### 2.1. Intestinal Barrier

Basically, the spatial interaction between microbiota and intestinal immune system can be divided into three layers. The first layer, facing to the intestinal lumen, is composed mainly by mucus and can be divided into another two sublayers: the outer sublayer, less dense, is highly colonized by microbiota, while the inner mucous layer is composed of high concentration of bactericidal antimicrobial peptides (AMPs) and secretory IgA (SIgA) specific for commensals microorganisms. Due to these components, the inner dense layer is virtually impervious to microbes [39–41].

The second layer is composed of a monolayer of intestinal epithelial cells (IECs) that are in touch with the lamina propria (LP) in their basolateral surface and with the mucous layer in their apical surface. The IECs are composed by several cellular types, like goblet cells which produce mucin (forming mucus); absorptive enterocytes and enteroendocrine cells, both producing cholecystokinin and ghrelin (which regulate appetite); Paneth cells, the leading producer of AMPs; and M cells, involved in capturing antigens to present them to immune system [42, 43]. IECs have a very important role in separating the internal body organs from the outside environment through the formation of tight junctions and secretion of mucus and AMPs (such as defensins, lysozymes, cathelicidins, phospholipase-A2, and C-type lectins) [42]. Furthermore, ECs express pattern-recognition receptors (PRRs), which include Toll-like receptors (TLRs), Nod-like receptors (NLRs), and Rig-I like receptors [44]. Interestingly, the production of some types of AMPs, like regenerating islet-derived protein 3*γ* (REGIII*γ*), REGIII*β*, and angiogenin-4, is influenced by commensal microorganisms in a TLR/MyD88 dependent way. However, other AMPs like lysozyme, phospholipase-A2, and defensins seem not to be influenced by microbiota [42]. A very important cell type present in IECs layer is the M cells. These cells work directly with the immune system, sampling antigens from lumen and carrying them in a unidirectional way to antigen presentation cells localized under the epithelium [42]. Enteroendocrine cells also act in gut barrier protection by producing enteroendocrine peptide glucagon-like peptide-2 (GLP-2), which is regulated by the nutritional status of the host, such as short-chain fatty acids production. The main characteristics in gut barrier function of GLP-2 are inducing intestinal epithelial cell proliferation; increasing the expression of intestinal tight junction proteins; and regulating the innate immune system by controlling the expression of antimicrobial peptides produced by Paneth cells [45].

The third layer, under the IECs, is formed by lamina propria and mesentery. The elements of the local immune system denominated gut-associated lymphoid tissues (GALT) are located within this layer. In the lamina propria, mature isolated lymphoid follicles (ILFs), which are formed from crypt patches (prenatal) and Peyer's patches (PPs), can be found. Microbe-associated molecular patterns (MAMPs) derived from colonizing bacteria are sensed by PRRs on IECs or dendritic cells (DCs) that recruit and activate T and B cells in ILFs. PPs, under IECs, receive antigens through M cells and pass them to DCs, which interact with T and B cells. In PPs and ILFs there are several plasma cells that normally produce and release IgA. DCs that sample antigens from LP or through IECs migrate to mesenteric lymph node to induce differentiation of effector T cells that traffic to the lamina propria [46].

### 2.2. Gut Microbiota and Intestinal Immune System Interaction

**Figure 1:**
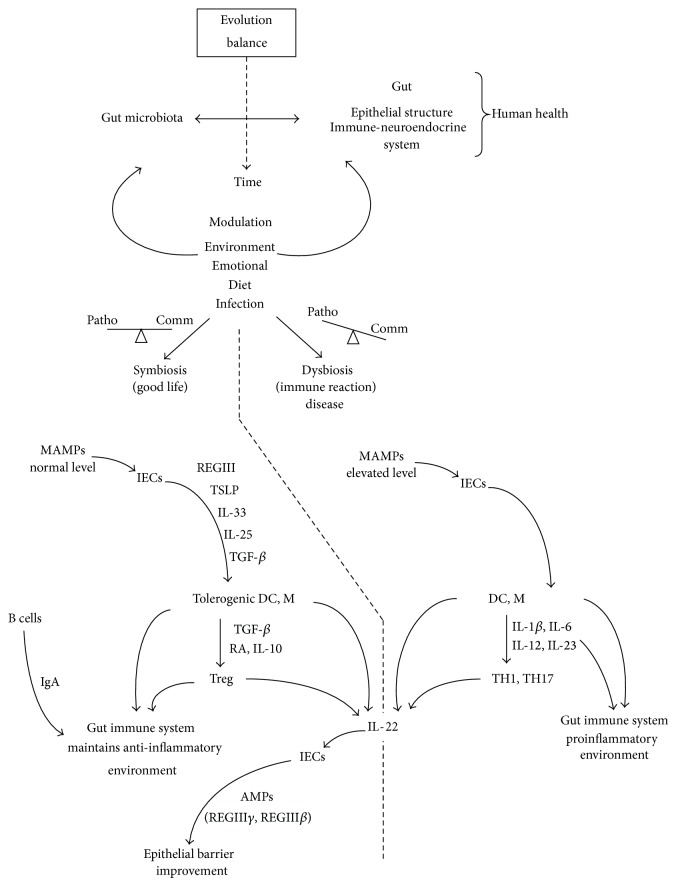
The functional interaction between microbiota and intestinal immune system. The evolutionary balance is formed over time, being modulated by the environmental pressure. Gut microbiota and gut environment are developed together, fitting for the benefit of both or tolerating each other. The immune system monitors the interaction to ensure homeostasis and contributes to symbiosis. However, the unbalance caused when dysbiosis is installed may cause the immune system reaction. Symbiosis and dysbiosis depend on balance between commensal and pathogenic bacteria. Commensal bacteria promote an anti-inflammatory environment. In a symbiosis context, MAMPs continuously stimulate IECs to secrete molecules that act protecting the epithelium and producing a tolerogenic environment. In dysbiosis, there is a significant liberation of MAMPs that can induce IECs, activated DCs, and macrophages to secrete inflammatory cytokines. Consequently, a development of immune effectors is generated. IL-22 is produced in both situations, but its contribution to epithelial barrier improvement is controlled by immune regulation. M: macrophage; Comm: commensal bacteria; Patho: pathogenic bacteria.

The functional interaction between microbiota and intestinal immune system begins with commensal bacteria that promote an anti-inflammatory environment (this process is summarized in Figure 1 and in the text below). In a symbiosis context, MAMPs continuously stimulate IECs to secrete regenerating REGIII*γ* into the lumen, thymic stromal lymphopoietin (TSLP), IL-33, IL-25, and tumor growth factor-*β* (TGF-*β*) under epithelium. These immunological mediators induce the development of tolerogenic macrophages and tolerogenic DCs [39, 46]. Tolerogenic DCs produce TGF-*β* and retinoic acid (RA) that stimulate the development of T regulatory cells. Thus, through Treg cells (that use diverse mechanisms of regulation), macrophages (that produce IL-10), and tolerogenic DCs, the gut immune system is able to establish and maintain an anti-inflammatory environment. In addition to essential regulatory roles of TGF-*β*, this cytokine is associated with other epithelial-derived substances (such as B-cell activating factor (BAFF) and proliferation-inducing ligand (APRILL)), in order to induce development of IgA-producing cells (plasma cells) [47]. This immunoglobulin is able to prevent the binding of commensal bacteria on host epithelium and is thus involved in the formation of the gut microbiota [48].

In a dysbiosis context, the presence of the pathogens can disrupt this regulated anti-inflammatory environment. When enteric pathogens overcome commensal bacteria, the imbalance between commensal and pathogenic bacteria causes a significant liberation of MAMPs. This increase in MAMPs can induce IECs, activated DCs, and macrophages to secrete inflammatory cytokines like IL-1*β*, IL-6, IL-12, and IL-23. These cytokines stimulate the development of effector CD4^+^ T helper 1 (TH1) cells and TH17 cells (that produce IL-17A, IL-17F, and IL-22) resulting in chronic inflammation [39]. In this context, the IL-22 cytokine has a crucial role. This molecule, produced by TH17 cells and by innate immunity cells (like NK-cells and *γδ*T cells), acts on intestinal epithelial cells by inducing the expression of several AMPs as REGIII*γ* and REGIII*β* that directly affects the microbiota. Interestingly, activated proinflammatory cells seem to work both in symbiosis and in dysbiosis; however, in case of symbiosis, the proinflammatory cells are kept under control by regulatory mechanisms (tolerogenic DCs and macrophages and T regulatory cells) and contribute by releasing IL-22, which promote production of REGIII*γ* by IECs and help to protect the epithelial barrier [39].

Although the mechanisms above described are already well established and despite of the existence of a vast literature about the subject, many aspects of microbial/immune system relationship still need to be elucidated. Furthermore, recent studies have added further evidence that demonstrate how the microbiota and immune system can interact to maintain homeostasis. Thus, the next paragraphs will describe some of the new evidences supporting this idea.

### 2.3. New Evidences about Gut Microbiota and Intestinal Immune System

Other recent studies have addressed the interactions between the gut microbiota and the immune system. These interactions may be related to maintaining the balance between the gut microbiota and immune system axis, both local and systemic.

Masahata et al. [49] showed the existence of a relationship between the IgA-secreting cells and the microbiota composition. In this study, to assess the importance of appendix associated lymphoid tissue (called caecal patches) in IgA-secreting cells generation, germ-free mice were appendectomized and colonized with bacteria. These authors found a decrease in IgA-secreting cells in large intestine, as well as a reduction of faecal IgA levels. Concomitantly, a significant reduction in the number of faecal bacterial species in appendectomized mice was noticed. However, in a very interesting way, these differences in the number of IgA-secreting cells and bacterial community disappeared after eight weeks of colonization. This normalization of colonic IgA-secreting cells correlates to increasing and enlargement of the solitary intestinal lymphoid tissues. Thus, these results suggest that IgA-secreting cells are involved with the maintenance of microbial homeostasis in the large intestine and contribute to shaping of the normal microbial community. Moreover, these findings demonstrate that development of immune system and microbiota are in a close accordance.

Forkhead box P3 (FoxP3) regulatory T cells (Tregs) perform an important role in gut homeostasis, mainly by controlling the function and proliferation of effector T cells. Several works have already demonstrated that germ-free mice are defective in these cells, proving a crucial role of the microbiota on Treg induction [50, 51]. Recently, Cording et al. [52] evaluated the commensal microbiota influence in proliferation of T CD4^+^ cells and Treg cells in animals submitted to long-term antibiotic treatment. These studies showed a significant reduction in the number of Treg cells on mesenteric lymph nodes and Peyer patches after treatment. Treg cells proliferation was also reduced in these tissues but not in the spleen and peripheral lymph nodes. Interestingly, the microbial reduction affected the proliferation of conventional T CD4^+^ cells in all analysed tissues (mesenteric lymph nodes, Peyer patches, peripheral lymph nodes, and spleen). Thus, the authors conclude that microbial stimulus locally affects the Tregs proliferation while conventional CD4^+^ T cells are affected systemically. This study, together with several others, confirms microbiota influence in homeostasis through Treg formation.

Despite the undoubted influence of microbiota in the regulatory cells, the mechanisms by which bacterial population induces the development of Treg cells remain poorly understood. To unravel this mystery, Obata et al. [53] inoculated germ-free mice with commensal microbiota and monitored the changes in IL-2 expressing-CD4^+^ T cells and FoxP3^+^ Treg cells population in lamina propria. The results showed an increase on IL-2^+^ CD4^+^ T cells that peaked in day 3 of bacterial colonization and returned to basal frequency around day 7. However, the analyses of the kinetics of Treg cells expansion demonstrated that, different from IL-2^+^CD4^+^ T cells, Treg cells continued to expand and became the most abundant CD4^+^ T cells in colon. This expansion was dependent on early IL-2, considering that treatment with neutralizing antibody to IL-2 abrogates this event. These findings suggest that microbiota stimulated the Treg cells development in an IL-2 dependent manner.

After determining the importance of IL-2, this study compared the genes that are upregulated in Treg cells responsive to IL-2. These comparisons allowed selecting the Uhrf1 gene (“ubiquitin-like, with pleckstrin-homology and RING-finger domains 1”) that was upregulated in colonic Treg cells. In agreement,* Uhrf1* knockout mice showed a defective accumulation of colonic Treg cells that was associated with spontaneous development of colitis. Thus, the authors suggest that colonizing bacteria can elicit, through antigen presentation cells, an early IL-2 production by effector CD4^+^ T cells. This IL-2 provides a signal for Tregs proliferation and to induce upregulation of* Uhrf1* gene. This last event supports the continuous proliferation of Treg cells that are able to prevent excessive immune response against microbiota.

Attempting to determine how commensal microbes can regulate host intestinal immunity and promote homeostasis, Mortha et al. [54] performed a very interesting and important work that established an axis between microbiota, innate immunity, and regulatory cells. Evaluating the role of granulocyte-macrophage colony-stimulating factor (GM-CSF)—renamed colony-stimulating factor 2 (Csf2)—in intestinal homeostasis, the authors observed that Csf2 knockout mice (Csf2^−/−^) presented a significant reduction in the frequency, number, and proliferation of regulatory cells (CD45^+^ TCR*β* ^+^ CD4^+^ FoxP3^+^) in the colon. These alterations in Tregs number were associated with a significant reduction in the frequency and number of IL-10- and IL-2-producing cells and with an increase of colonic IFN-*γ* producing T cells. Moreover, in Csf2^−/−^ mice were found reduced numbers of colonic dendritic cells (DCs) and macrophages besides a significant reduction in production of regulatory mediators (retinoic acid, IL-10, and TGF-*β*) important to Treg cells generation. These results demonstrate that Csf2 is involved in colonic homeostasis influencing the number, frequency, and function of DCs and macrophages and, thereafter, in Treg differentiation.

Once the importance of Csf2 for homeostasis is known, the study showed that ROR*γ*t^+^ type 3 innate lymphoid cells (ILC3) (reviewed in [55]) localized in isolated lymphoid follicles (ILFs) are the main producers of Csf2 and this production is stimulated by macrophage-derived IL-1*β*. Finally, using antibiotic treated mice and MyD88 knockout mice, this work determined that the microbiota is able to stimulate the macrophage-derived IL-1*β* production in a MyD88 dependent way. Collectively, these results revealed that commensal bacteria are sensed by macrophages that produce IL-1*β*. This cytokine stimulates the release of Csf2 by ILC3, which in turn controls the production of regulatory mediators by DCs and macrophage, to maintain colonic Treg homeostasis. Disturbance in this relationship induces homeostasis breakdown and can result in impairment of oral tolerance to dietary antigens [54].

Several works are trying to identify metabolites of the microbiota able to influence the immune system and induce homeostasis. In this context, Smith et al. [56] demonstrated that germ-free mice have significant reduction on the concentration of three of the most abundant types of short-chain fatty acids (SCFA: acetic acid, propionic acid, and butyric acid) suggesting a relation between these molecules and the immunological problems faced by this kind of mice. To clarify this question, germ-free mice were treated with SCFA (individually or in combination) for 3 weeks. As expected, these mice showed increase in frequency and number of colonic Treg cells, which do not happen with TH1 or TH17 cells. The SCFA treatment was also able to induce increase of* FoxP3* and* IL-10* gene expression and IL-10 production, suggesting that SCFA can induce specifically FoxP3^+^ IL-10-producing Treg cells. Moreover, the SCFA treatment was able as well to reduce the symptoms of T cell-transfer model of colitis. Collectively, these results demonstrate that SCFA play an important role in maintaining homeostasis through Treg cells.

The actions of microbiota-derived metabolites on Treg cells (mainly SCFA) were also confirmed by other studies conducted by Furusawa et al. [57] suggesting that these compounds can subvert the adaptative immunity, diminishing the effector response and contributing to health.

The consequences of losing the intestinal immunologic control are not merely local but do reflect in a systemic manner. The lack of homeostasis may lead to invasion of immunogenic molecules derived from the cell wall of Gram-negative bacteria to the bloodstream, in a condition named endotoxemia. Changes in gastrointestinal barrier function, caused by diet change, can also develop endotoxemia [58]. The increase in gut permeability can be caused by alterations in the gut microbiota; alterations in the expression, localisation, and distribution of tight junction proteins (claudin, ZO-1, and occludin); decrease in intestinal alkaline phosphatase activity leading to a decrease in LPS detoxification; and, recently observed, overactivation of the CB1 receptor (discussed later) [59]. During dysbiosis, the gut microbiota may produce high levels of endotoxins, which once in the bloodstream cause mild and continuous induction of proinflammatory mediators, resulting in low-grade systemic inflammation. This inflammatory state contributes to the progression of many human diseases, including obesity, type 2 diabetes, liver and cardiovascular diseases, and inflammatory bowel diseases.

In order to visualize how the microbiota influences the immunologic status as a whole, IBD is given as an instance, as it is one of the most studied diseases and one of the most aggressive conditions related to the gut microbiota and immune system. Numerous studies have correlated the reduction in* Faecalibacterium prausnitzii *(which belongs to the phylum Firmicutes and is the major bacterium of the* Clostridium leptum* group) to IBD. Cao et al. [60] by meta-analysis (with a total of 1180 patients analyzed) revealed that IBD patients have a significant reduction of* F. prausnitzii*. The authors suggest a possible protective benefit of* F. prausnitzii* against the development of IBD and recommend the use of prebiotics and probiotics so as to augment the levels of this species.  Table 2 summarizes more examples of the immune alterations which happen due to alterations in the levels of specific bacteria.

As demonstrated by the studies described above, the intestinal microbiota and the immune system interact continuously to the establishment of a complex dynamic equilibrium that maintains the host health. Despite numerous papers that address this issue, many gaps remain to be elucidated and several other strategies will be needed to answer these questions. Nevertheless, a complete understanding of the immunity/microbiota relationship may be the key to treatment of several important diseases that affect humans.

## 3. Gut Microbiota and Neuroimmune System Interaction

Microbiota can alter behavior, humor, and anxiety in stress response [61]. These alterations can be achieved through the pituitary-adrenal axis (HPA) system. Several researches have demonstrated by distinct methodologies, such as germ-free mice [62], pathogenic bacteria infection [63], antibiotic use [64], vagotomy [65], and measurement of excitation by vagal afferents [66], a role for enteric nervous system (ENS) and vagus nerve, which belongs to the autonomic nervous system (ANS), as pathways for modulating the central nervous system (CNS) by microbiota. Inversely, they also demonstrate how CNS or ANS influence microbiota via intestinal secretion and motility, besides the soluble molecules in the lumen and internally below the gut epithelial layer. In addition to this, there is hormone releasing by epithelial cells and secreted microbial products that induce the epithelial releasing of molecules that modulates the neural system [69]. To understand this systemic communication branch, it is necessary to understand the two main gut-brain axes: the HPA and the ANS.

HPA axis initiates with the secretion of corticotropin-releasing hormone (CRH) by neurons in the paraventricular nucleus of the hypothalamus. CRH reaches the anterior portion of pituitary gland, which secretes adrenocorticotropin hormone (ACTH) into the bloodstream reaching the adrenal glands and inducing cortisol release that will act throughout the body via glucocorticoid receptor (GR). This phenomenon was named adaptive stress response [67, 68].

ANS is divided into sympathetic, parasympathetic, and enteric systems. To detect the signals generated in the gut, the ANS make use of sensory neurons that are divided into intrinsic ones localized inside the tissular intestinal structure, as the intrinsic primary afferent neurons (IPANs), which are located in the myenteric nervous system, and extrinsic ones that comprise the vagal and spinal extrinsic primary afferent neurons, which are out of the tissue structure of the intestine and project dendrites to form synapses with the enteric neurons. To complete the neuronal intestinal network, sympathetic neurons communicate with the myenteric plexus, by innervating each other [69].

Vagus nerve provides information from intestines to the brain by solitary tract and sends information that can alter behavioural responses by activation of the interaction between locus coeruleus, also considered a major site for integrating stress responses, and forebrain, to produce corticotropin-releasing factor (CRF) [70, 71]. In addition, vagus nerve talks to the hypothalamus interfering in the HPA axis [72]. This chronically activated pathway promotes neural alterations leading to anxiety, panic disorders, and depression [73]. This view brings ideas for investigating the cross talk between gut bacteria and the CNS via vagus nerve and HPA axis.

### 3.1. Gut Microbiota and HPA Axis

It has been reported that HPA axis prevents massive damage to the inflammatory sites. Once the stress response is activated, cortisol secretion negatively regulates inflammation and immune response. Overactivation of HPA axis by chronic stressors may explain its detrimental effects on immune cells [67].

For example, while in mast cells cortisol inhibits the release of histamine, which reduces eosinophil recruitment, in T cells the glucocorticoid receptors regulate the expression of IL-4, IL-5, and IL-13 when exposed to allergens [74, 75]. It has been proved that not only the brain but also immune cells are sources of neuropeptides. Kavelaars et al. [76] showed that corticotropin-releasing factor and arginine vasopressin can induce secretion of beta-endorphin in mononuclear cells. Moreover, Westly et al. [77] provided strong evidence that immune cells can synthesize proopiomelanocortin. In addition, glutamate is known to be produced by dendritic cells (DCs) in the context of antigen presentation [78]. Literature has increased when regarding neuroactive products being endogenously produced by immune cells (Table 3) [78–86].

Most importantly, little is known about the idea of microorganisms or their products to be responsible for triggering the neuroactive components release by immune cells. Indirectly, it has been demonstrated that microbiota can program central responses. While germ-free mice had an overstressed response that could be reversed by microbiota reconstitution with faeces or with* Bifidobacterium infantis* [86], enteropathogenic* Escherichia coli* were capable of enhancing the response to stress.

Ait-Belgnaoui et al. [87] suggested that microbiota might alter gut permeability and lead to lipopolysaccharides (LPS) transmigration into the blood, increasing neuroendocrine response to stress. Probiotic treatment attenuated HPA response by enhancing the intestinal-epithelial barrier, thus reducing circulating LPS. It leads to the conclusion that gut bacteria have an important role in altering HPA response by acting directly with part of its structure or indirectly by protecting gut permeability. However, the underlying mechanisms remain unclear.

The opposite way also occurs. For example, mice exposed to a social stressor called social disruption presented significantly changed community structure with decreased abundance of* Bacteroides *spp. and increased* Clostridium* spp. In addition, increased circulating levels of IL-6 and the chemokine CCL2 (also known as MCP1) were shown, which is indicative of immune reaction [88].

### 3.2. Gut Microbiota and Development and Regulation of CNS

As we have seen, the gut microbiota influence is not restricted to the gastrointestinal tract, and studies show the close relationship between the microorganisms and the development and regulation of the nervous system [62, 75, 87]. This influence is due to the fact that microbes are capable of releasing products that act upon the development and function of the nervous system [61, 67, 68, 70]. In this context it is necessary to elucidate the beneficial and deleterious effects of the gut microbiota in the nervous system [43].

Recent studies demonstrated that morphological and functional abnormalities of the enteric nervous system (ENS), the complex neuronal network that autonomously regulates most gastrointestinal functions, also could be related with microbiota and immune system. Using TLR2 knockout mice (TLR2^−/−^), Brun et al. [89] detected a significant reduction in the number of enteric glial and neuronal cells in these mice, suggesting that the development of these cell types is dependent on TLR2 signalling. In addition, alteration of neurochemical profile (reduction of neuronal nitric oxide synthase—nNOS), increase of the frequency and amplitude of spontaneous contraction, elevation of intestinal traffic, and reduced levels of glial cell line derived neurotrophic factor (GDNF) in smooth muscle cells were observed. All these changes in TLR2 mice were completely reversed by administration of exogenous GDNF, confirming that these abnormalities on enteric nervous system are TLR2/GDNF dependent.

To investigate the influence of gut microbiota on ENS integrity and function, wild type mice (C57BL/6j) were depleted from microbiota through treatment with broad-spectrum antibiotics. These depleted mice presented reduced expression of neuronal peripherin, nNOS, and glial S100*β* proteins, similarly to TLR2^−/−^. All these alterations were associated with a reduction of GDNF expression and, again, the supplementation with GDNF was able to reverse these abnormalities. In a very interesting way, these defects presented by microbiota-depleted mice were also partially restored when these mice received TLR2 agonist. Thus, this work confirms that ENS integrity and functionality are dependent on gut microbiota and TLR2/GDNF pathway. Moreover, these results showed that microbiota stimulated-TLR2 not only represents an immunological role, but also influences directly ENS integrity and is very important to preserve gut homeostasis [89].

IPANs, in the myenteric plexus of the enteric nervous system, provide the intestinal mucosa with sensory fibers that innerves the gut velocities [90, 91]. In this regard, IPANs are neurons cells prone to respond to probiotics and commensal bacteria and alter the gastrointestinal physiology [69]. As they are also sensitive to bioactive bacteria and to neurotransmitters released by microbes, Kunze et al. [92] verified that rats fed with* Lactobacillus reuteri *displayed increased excitability and number of action potentials in IPANs. Other studies showed the analgesic activities promoted by species from the* Lactobacillus *genus, which was obtained from the inactivated microorganism and conditioned media used [93, 94].

The* Lactobacillus reuteri* CRL1098 and JCM1112, isolated from the human intestine and other animals, can produce vitamin B12, an important vitamin for the nervous system, and its deficiency could induce neuropathies [62]. Wall et al. [95] demonstrated in their study that when* Bifidobacterium breve* strains, a commensal group, were used as probiotic, the mice brain fatty acid composition was changed, showing increase in concentration of arachidonic and docosahexaenoic acid, both important in neurogenesis and neurotransmission, when compared to the nonsupplemented group.

Taylor and Feng [96] showed that circulating substances in the blood, such as tryptophan (an important precursor of the neurotransmitter serotonin), are changed with the presence or absence of intestinal microbiota. Treatment of Sprague-Dawley rats with* Bifidobacterium infantis* shows an increase in plasma tryptophan and decrease in frontal cortex 5-hydroxyindole acetic acid (5-HIAA) levels, which suggests that there may be happening reduced serotonin degradation in this brain area. Moreover, the supplementation with* Bifidobacterium infantis* was capable of reducing the inflammatory mediators (IL-6, TNF-*α*, and IFN-*γ*), demonstrating the influence of gut microbiota also on the immune system [97]. The increase of tryptophan is consistent, once IFN-*γ* has been shown to be a potent stimulus in the activation of indoleamine (2,3)-dioxygenase (IDO), the enzyme involved in the conversion of tryptophan to kynurenine [96].

### 3.3. Gut Microbiota and Experimental Autoimmune Encephalomyelitis Model

Taking into account the relationship between the nervous system, the immune system, and the gut microbiota, it is important to highlight studies that relate the influence of these microorganisms in the development of autoimmune diseases, as multiple sclerosis, directly related to the CNS [43].

Experimental autoimmune encephalomyelitis (EAE) is an experimental model used to study multiple sclerosis, an autoimmune demyelinating disease of the central nervous system. Although the cause of the disease remains unknown, studies have reported the involvement of environmental factors associated with a genetic predisposition. The immune response in EAE is mainly characterized by T helper 1 and T helper 17 cells [98]. Segmented filamentous bacteria (SFB) present in the intestine are related to the induction of Th17 and are indicated to be associated with autoimmune diseases with such cellular profile [99].

In order to verify the influence of intestinal microbiota on the development of EAE, induced animals were treated with antibiotics to reduce the intestinal microbiota; the results showed reduction of clinical signs of EAE in animals with compromised gut microbiota; this reduction was accompanied by a decrease of IFN-*γ*, MIP-1*α*, MIP-1*β*, MCP-1, IL-17, and IL-6 associated with increased IL-10 and IL-13 release [69]. Ochoa-Repáraz et al. [100] relate the action of B CD5^+^, regulatory B cells, to this improvement of clinical signs in microbiota altered by antibiotics.

The use of germ-free mice also demonstrates the importance of the intestinal microbiota in the development of EAE. Clinical signs of EAE in germ-free animals are attenuated when compared to conventionally colonized animals. These animals showed reduction in the production of proinflammatory cytokines in the CNS, accompanied by increase in number of regulatory cells. Lee et al. [101] induced EAE in germ-free animals and observed a reduction of the inflammatory cytokines (IFN-*γ*, Il-17A) together with an increase in the regulatory T cells CD4^+^CD25^+^ FoxP3^+^ (Tregs) not only in the gut, but also in the spinal cord when compared to the wild type mice. Additionally, deficiency was found in dendritic cells to promote differentiation of TH17 cells in germ-free mice [69, 101].

Research in this area is still very incipient and not conclusive. As above described, evidences from works involving EAE indicate that the benefits brought by the microbiota do not apply to improving symptoms of this model. However, recent findings showed that specific microorganisms of the intestinal microbiota could improve the clinical signs of EAE. In this case, these strains of lactobacilli can enhance the immune-regulatory activity both by increased production of IL-10 and by increased rate of B and T regulatory cells. In this study it was found that, of the three strains used, monostrain oral treatment failed therapeutically in EAE, and mixture of lactobacilli strains suppressed the progression and reversed the clinical and histological signs [102].

Thus, the interaction of gut microbiota, immune system, and nervous system is not fully understood with many points remaining to be clarified, which justifies the development of new studies.

### 3.4. Gut Immune System and Nervous Cannabinoid Signaling

Recently a novel signalling pathway correlating gut immune system and nervous cannabinoid receptors has been investigated. As well known, cannabinoids receptors, composed by CB1 and CB2 receptors, are present in immune and neural cells [103–105]. Recently CBs were found in the luminal surface of the epithelial microvilli, Peyer's patches, ganglionic cells of the myenteric plexus, and smooth muscle of the blood vessels walls [106]. The localization of these receptors in the intestinal epithelium, immune system cells, and nervous system brings new perspective on treatments of disorders related to those systems. From what is already known, CB2 receptors have been connected to analgesic and anti-inflammatory functions in several experimental models of colitis [107, 108].

Such field has gained attention since Rousseaux et al. [94] showed increased mRNA expression of receptors CB2* in vitro* and in epithelial cells in the colonic section after oral administration of* Lactobacillus acidophilus* NCFM. This result was accompanied by decrease in normal visceral perception. The improvement of visceral pain was attributed to direct contact of NCFM with epithelial cells able to induce CB2 expression, through the NF-*κ*B pathway. Recently, Aguilera et al. [109], after causing dysbiosis by stress and antibiotic treatment, showed increased CB2 receptor mRNA expression in colonic tissues of mice. During the investigation, the authors found increased CB2 expression to be positively correlated with* Lactobacillus* spp. counts and negatively correlated with* Clostridium* spp. counts. Those observations indicated that intestinal endocannabinoid system might modulate visceral pain response and the presence of a bacterial group as a pathogenetic component [109].

Karmaus et al. [110] verified that CB1 activation, by gut microbiota, increased gut permeability. This permeability is caused by altering the distribution of tight junction proteins which elevates endotoxemia. The use of prebiotics for regulating gut microbiota or antagonist of CB1 in obese mice models regulated gut permeability with improved distribution and localisation of tight junction proteins.

Once CBs receptors of intestinal tissue are activated by cannabinoids ligands, it may also activate CBs receptors of other local systems. These data begin to become interesting when they are crossed with studies about cannabinoid system of immune cells. Karmaus et al. [110] demonstrated that the CB1 and CB2 knockout DC presented augmentation of activation-related molecules, such as MCH I, MHC II, CD80, and CD86, after contact with LPS. Chiurchiù et al. [105] verified that the treatment with anandamide, an endocannabinoid, on DCs isolated from healthy donors and multiple sclerosis patients, led to a decrease on production of TNF-*α* and IL-6. In the same study, it was also shown that treating the DCs with anandamide also decreased their ability of inducing Th1 and Th17. An increase in CB2 expression accreted of decrease of fatty acid amide hydrolase (anandamide degrading enzyme) was also noted in the multiple-sclerosis-patient DCs in comparison to DCs isolated from healthy donors. Such evidences support the DCs to be immunomodulated by cannabinoids. Furthermore, the immunomodulation of DCs by eCBS follows stimulus and polarization of the T cells. Thus, a new possible interaction between gut microbiota and immune system can be perceived through regulation by the endocannabinoid system, having as an initial aim multiple sclerosis studies, as well as an opportunity to understand the interaction comprised in the axis gut-immune-brain.

## 4. Conclusion

The intestinal microbiota has drawn progressively more attention from the scientific community due to the association of its role in the human physiology and in the development of diseases following dysbiosis. It is known to be associated with regulation of digestion, absorption of nutrients, biochemistry processes, immune modulation of the mucosa, and the production of toxins substances, autonomous nervous system interaction, and nervous development. In order to advance in the understanding of this complex interaction, the screening of the possible interactions of metabolic pathways is made necessary. Taking a beneficial view of prebiotic and probiotic, mapping the microbiome in agreement with nutrigenomics and nutrigenetics may give rise to the construction of nutritional metabolic collections. These research areas might potentially aid in unraveling several hypotheses related to ambient factors that may lead to disorders of unknown etiology such as the hygiene hypothesis, which postulates that decreased microbial exposure has, in part, driven immune deregulation. Further studies are still needed in order to clarify the interaction between gut microbiota and neuroimmune system, as well as with endocrine system, so as to create nutrigenetic profiles that may aid in reaching individual homeostasis.

## Figures and Tables

**Table 1:** Profile of alterations in the gut microbiota in IBS, IBD, colorectal cancer, obesity, and type 2 diabetes.

Disease	Microbial alteration	Reference
Irritable bowel syndrome	Increased presence of Firmicutes, specifically *Ruminococcus *sp*., Clostridium *sp., and* Dorea *sp.; reduction in *Bifidobacterium* and *Faecalibacterium *spp.; decrease of *Bacteroides* in afflicted children; increased presence of *Dorea *sp*., Ruminococcus *sp., *Haemophilus *sp. and *parainfluenzae *sp. *in paediatric patients.*	[9–11]

Inflammatory bowel disease	Reduced complexity of Firmicutesand Bacteroidetes,* with decrease in the abundance of Clostridium leptum* and *Clostridium coccoides;* increase in bacteria of the Gammaproteobacteriaclass*;* presence of adherent and invasive *Escherichia coli;* decreased presence of *Faecalibacterium prausnitzii;* altered abundance of members of the families Enterobacteriaceae, Ruminococcaceae, and Leuconostocaceae,with increased presence of *Clostridium *and reduced presence of *Roseburia *and *Phascolarctobacterium. *	[12–14]

Colorectal cancer	Members of the genus *Fusobacterium *appear increased on colorectal cancerous tissue; reduction in bacteria of the phyla Firmicutesand Bacteroidetes; alterations in number of butyrate producing bacteria (*Coprococcus *spp.;* Eubacterium rectale*; *Roseburia *spp.;* and Faecalibacterium prausnitzii),* related to the protective effect of butyrate for the enterocytes.	[15, 16]

Obesity	Decreased presence of Bacteroidetes; increased presence of Actinobacteria.	[3, 17]

Type 2 diabetes	Overall alterations of the microbiota; increased presence of *Clostridium *spp.; *Akkermansia muciniphila; Bacteroides* spp.; and *Desulfovibrio* spp.	[18]

Ulcerative colitis	Decreased presence of Firmicutes, Lentisphaerae, and Verrucomicrobia*;* increased presence of Proteobacteria, Fusobacteria, and Spirochaetes.	[19]

**Table 2:** Gut microbiota microorganisms and correlated immune state, disease, or symptoms.

Gut microbiota microorganism	Model system studied	Associated physiopathological condition	References
*Bifidobacterium lactis* (LAFTI B94)	Rat	Decrease in the levels of TNF and iNoS in rats with colitis induced by TNBS	[20]
*Bifidobacterium infantis* (35624)	Mouse	Induction of Treg and inhibition of NF-*κ*B in mice with enteric *Salmonella-*induced enteritis	[20]
*Escherichia coli* (Nissle 1917)	Human and mouse	Diminishing of TLR2- and TLR4-induced inflammation of the colon in humans and mice with ulcerative colitis and colitis induced by DSS	[20]
*Lactobacillus rhamnosus* (Lr32 and GG)	Mouse and rat	Induction of Treg in mice and rats with colitis induced by TNBS associated with hLA-B27	[20]
*Lactobacillus salivarius* (Ls33)	Mouse	Decrease of colonic inflammation of mice with colitis induced by TNBS	[20]
*Lactobacillus reuteri* (strain not specified)	Mouse	Upregulation of NGF and decrease of IL-8 and TNF levels in IL-10 deficient mice	[20]
*Lactobacillus plantarum* (299V)	Mouse	Decreased levels of IFN-*γ* and IL-12p40 in IL-10 deficient mice	[20]
*Lactobacillus fermentum* (CECT5716)	Rat	Lower levels of TNF and iNoS in the colon of rats with colitis induced by TNBS	[20]
*Lactobacillus casei* (LAFTI L26)	Rat	Decreased levels of cyclooxygenase 2 in the colon of rats with TNBS-induced colitis	[20]
*Lactobacillus acidophilus* (NCFM)	Human	Prevention of the loss of insulin sensibility in individuals with glucose intolerance and/or diabetes mellitus	[21]
*Lactobacillus gasseri* (SBT2055)	Human	Weigh, BMI, circumference of waist and hip, and visceral and subcutaneous fat reduction in individuals with BMI from 24,2 to 37 km/m^2^ and visceral fat accumulation	[21]
*Bacteroides thetaiotaomicron* (strain not specified)	Rat	Decrease in the levels of IL-8 and TNF in rats with enteritis induced by enteric *Salmonella *	[20]
*Bacteroides fragilis* (wild type)	Mouse	Production of IL-10 derived of T CD4+ in mice with colitis induced by TNBS	[20]
*Fusobacterium nucleatum* (ATCC 25586)	Human	Occurrence of colon-rectal carcinoma	[22]
*Faecalibacterium prausnitzii* (DSM 17677 in mouse and wild type in humans)	Human and mouse	Decrease in the levels of NF-*κ*B, IL-8, and TNF and increase in the production of IL-10 in mice with TNBS-induced colitis; protection against development of IBD in humans	[20]
*Helicobacter pylori* (absent or present in low levels—wild type)	Human	Paediatric asthma and reflux esophagitis occurrence	[22, 23]
*Akkermansia muciniphila* (ATCC BAA-835)	Mouse	Improved metabolic disorders in diet-induced obese mice and counteracted diet-induced colon mucosal barrier dysfunction	[24, 25]

DSS, sodium dextran sulphate; IFN-*γ*, interferon-*γ*; IL, interleukin; iNoS, inducible nitric oxide synthase; NF-*κ*B, nuclear factor *κ*B; NGF, neural growth factor; TGF*β*, transforming growth factor-*β*; TLR, Toll-like receptors; TNBS, trinitrobenzenesulfonic acid; TNF, tumor necrosis factor; Treg, regulatory T cells.

**Table 3:** Cellular sources of neuroactive products in the immune cells.

Cellular source	Hormone/neurotransmitters
Lymphocytes	Acetylcholine, melatonin

B lymphocytes	ACTH, endorphins, GH, IGF-1

T lymphocytes	5-HT, ACTH, endorphins, TSH, chorionic gonadotropin, GH, PRL, parathyroid-hormone-related protein, IGF-1, VIP

Macrophages	ACTH, endorphins, GH, substance P, IGF-1, atrial natriuretic peptide

Dendritic cells	Glutamate, dopamine

Splenocytes	LH, FSH, CRH, adrenaline, endomorphins

Thymocytes	CRH, LHRH, AVP, OT, adrenaline

Mast cells	VIP, somatostatin

Neutrophils	VIP, somatostatin

Megakaryocytes	Neuropeptide Y

5-HT, 5-hydroxytryptamine (serotonin); ACTH, adrenocorticotropic hormone (corticotropin); AVP, arginine vasopressin; CRH, corticotropin-releasing hormone; FSH, follicle-stimulating hormone; GH, growth hormone; IGF-1, insulin-like growth factor 1; LH, luteinizing hormone; LHRH, luteinizing-hormone-releasing hormone; OT, oxytocin; PRL, prolactin; TSH, thyroid-stimulating hormone; VIP, vasoactive intestinal peptide.
